# Sarcoid Uveitis: An Intriguing Challenger

**DOI:** 10.3390/medicina58070898

**Published:** 2022-07-04

**Authors:** Pia Allegri, Sara Olivari, Federico Rissotto, Roberta Rissotto

**Affiliations:** 1 Uveitis and Eye Inflammatory Diseases Referral Center, Rapallo Hospital, 16033 Genova, Italy; sara.olivari@asl4.liguria.it; 2 Department of Ophthalmology, IRCCS Ospedale San Raffaele, University Vita-Salute San Raffaele, 20132 Milan, Italy; federico.rissotto@hsr.it; 3 Eye Clinic, San Paolo Hospital, University of Milan, 20142 Milan, Italy; roberta.rissotto@unimi.it

**Keywords:** sarcoidosis, ocular sarcoidosis, multimodal imaging, granulomatous uveitis, sarcoidosis-associated uveitis, ocular sarcoidosis diagnosis, ocular sarcoidosis therapy

## Abstract

The purpose of our work is to describe the actual knowledge concerning etiopathogenesis, clinical manifestations, diagnostic procedures, complications and therapy of ocular sarcoidosis (OS). The study is based on a recent literature review and on the experience of our tertiary referral center. Data were retrospectively analyzed from the electronic medical records of 235 patients (461 eyes) suffering from a biopsy-proven ocular sarcoidosis. Middle-aged females presenting bilateral ocular involvement are mainly affected; eye involvement at onset is present in one-third of subjects. Uveitis subtype presentation ranges widely among different studies: panuveitis and multiple chorioretinal granulomas, retinal segmental vasculitis, intermediate uveitis and vitreitis, anterior uveitis with granulomatous mutton-fat keratic precipitates, iris nodules, and synechiae are the main ocular features. The most important complications are cataract, glaucoma, cystoid macular edema (CME), and epiretinal membrane. Therapy is based on the disease localization and the severity of systemic or ocular involvement. Local, intravitreal, or systemic steroids are the mainstay of treatment; refractory or partially responsive disease has to be treated with conventional and biologic immunosuppressants. In conclusion, we summarize the current knowledge and assessment of ophthalmological inflammatory manifestations (mainly uveitis) of OS, which permit an early diagnostic assay and a prompt treatment.

## 1. Introduction

Sarcoidosis (S) is part of a heterogeneous family of granulomatous inflammatory diseases, triggered by one or more unknown antigens in predisposed hosts, causing non-caseating granulomatous inflammation. The clinical manifestations are polymorphous and frequently underestimated. Although the lung is usually the primary target of disease, S may affect any organ in the body, including the eye. Ocular involvement in S represents one of the leading causes of low visual acuity (VA), and sometimes blindness in affected patients [1]. Any part of the eye, adnexa, and orbit can be affected. However, anterior, intermediate, posterior, or pan-uveitis represent the most common forms of eye involvement, according to the standardization of uveitis nomenclature (SUN) [2]. Ocular symptoms might occur months before the systemic presentation of the disease, and they can present as a clinically isolated-to-the-eye condition. S diagnosis is often ignored when the patient first presents with ocular symptoms, and the disease is taken into consideration only when the subject suffers from other manifestations related to the multi-systemic characteristics of this disorder [3]. Thanks to recent improvements in diagnostic testing and treatment, ocular disease can be treated with a good visual outcome. However, if diagnosed later, it can progress until blindness.

The disease can present with a unilateral onset, but it then usually becomes bilateral, whether symmetric or asymmetric, and tends to chronicity. The most frequent form of OS is represented by anterior acute granulomatous uveitis. The main symptoms are photophobia, pain, redness, and blurred vision, although it may manifest as a chronic asymptomatic form, thereby causing a diagnostic delay which leads to irreversible ocular damage [4,5]. So far, only patients with a positive tissue biopsy (showing non-caseating granulomas) are classified as “definite” OS, according to the International Workshop on Ocular Sarcoidosis (IWOS) updated criteria (2019) [6,7]. Other diagnostic subgroups are classified as “presumed” and “probable” OS. The former is determined if diagnosis is not supported by biopsy but bronchoalveolar lavage (BAL) results are positive and two typical intraocular signs are present, while the latter is defined in case tissue biopsy and BAL are not available but two positive intraocular clinical signs and two systemic investigations are positive. Biopsy performance in uveitis consistent with diagnosis of S was found to be 4% in cases of normal chest CT and 70% if associated with hilar or mediastinal lymphadenopathy observed in high resolution chest CT and alveolar lymphocytosis [8].

Pulmonary diagnostics (chest X-ray, high resolution computed tomography (HRCT), bronchoalveolar lavage (BAL), ^67^gallium scintigraphy, ^18^FluoroDeoxyGlucose Positron Emission Tomography (^18^F-FDG-PET), mediastinal biopsy), central nervous system (CNS) investigations, namely, encephalic magnetic resonance imaging (MRI), and dermatological counseling can help make a correct diagnosis [9]. Concerning serological work-up, high levels of angiotensin converting enzyme (ACE), lysozyme, and ß-2 microglobulin define an active disease. Increased serum ACE has 83–95% specificity, however in healthy unaffected children ACE levels are normally high. Niederer et al. found that among 110 subjects affected by S, the median ACE was 97.0 (IQR 60.5–118.5) in adults, and 92.0 (IQR 58.0–100.5) in children. In healthy subjects the median ACE was 29.0 (IQR 20–40) in adults and 42 (IQR 28–57.5) in children [10].

Increased production of vitamin D from epithelioid cells causes hypercalciuria and, less frequently, hypercalcemia [9,10,11,12,13,14]. Cameli and colleagues studied a population of S patients and compared them to subjects suffering from idiopathic pulmonary fibrosis (IPF) and chronic hypersensitivity pneumonitis (cHP). Their work confirmed that changes in calcium metabolism, particularly hypercalciuria, occur frequently in patients with fibrotic S, supporting the hypothesis that an altered calcium metabolism may be a peculiar characteristic of sarcoid granulomas [15]. Other useful tests are the skin pathergy test and B lymphocyte polyclonal activation [16,17,18,19,20,21], which, however, were not analyzed in our patients.

Anterior granulomatous uveitis, mainly bilateral, is the typical presentation of OS, followed by intermediate uveitis with strings and pearls of vitreous inflammatory involvement predominantly at pars plana. It seldom presents as posterior uveitis with multifocal chorioretinitis, prevalently in old Caucasian females who may develop ocular and CNS complications [22].

Anterior segment slit-lamp examination shows perikeratic hyperaemia, granulomatous precipitates, posterior synechiae, and iris Koeppe (at the pupillary rim) or Busacca (at the level of iris stroma) nodules. Intermediate uveitis induces floaters and blurred vision as the main symptoms; fundus examination shows the typical string pearls of vitreous opacities, snowballs and snowbanks. Asymmetric bilateral posterior uveitis mostly presents with round-shaped choroidal granulomas that can be of different sizes and cause a reduction in visual acuity, especially if centrally located. Other frequent manifestations include periphlebitis, sectorial vascular sheathing, candle wax drippings, and cystoid macular edema (CME). Increased intraocular pressure is mainly present in anterior uveitic involvement and usually evolves towards severe glaucoma [23].

Ocular involvement is generally chronic, and patients suffer both from complications of the disease and from protracted therapy side effects; cataract can be the final complication of local or systemic steroid therapy and prolonged lack of treatment. The chief complications leading to surgery are cataract and glaucoma. Another frequent issue is CME, which requires frequently repeated intravitreal treatment, if resistant to local and systemic steroids or immunosuppressants [22]. Rarely, the course of ocular disease can be complicated by macular choroidal neovascularization (CNV) and retinal detachment due to pathological vitreous tractions [23].

OS diagnosis might be easier if it is associated with systemic manifestations, but when the ocular disease has a chronic inflammatory course, it can go unrecognized and untreated for years.

## 2. Literature Review

### 2.1. Epidemiology

Former epidemiological studies of S and OS were conducted more than 10 years ago and several of them were carried out without a precise biopsy-proven diagnosis; for this reason, the incidence of the disease can widely vary from 0.2% [24,25] to 0.8% [26] with unreliable results.

The average incidence ranges from 4.5 to 6.4% in tertiary referral centers [27,28,29,30,31,32,33,34,35,36,37,38,39]. Recent epidemiological studies by Cimino showed that OS accounts for 4.3% in the total analyzed uveitic population of a northern Italy tertiary referral center. Concerning sarcoid uveitis, anterior uveitis was present in 0.5%, intermediate uveitis in 2.7%, posterior uveitis in 4.3%, and panuveitis in 23% of the analyzed patients [39,40].

Other epidemiological data show that (a) the disease affects mainly African Americans (severe involvement) and Nordic European populations [41,42,43,44,45,46]; (b) the incidence in the USA ranges from 8.1/100.000 in Caucasians to 17.8/100.000 in African Americans [47]; (c) in Japanese patients [48] the incidence rate was 0.73/100.000 in males and 1.28 in females; (d) the prevalence was 4.5/100.000 in affected subjects in Northern Ireland [49].

Although S can affect anyone at any age, the onset is usually in adult age (between 20 and 50 years old) and children are rarely affected [50,51]. Another peak of first manifestation in Scandinavian and Japanese women was detected between 50 and 65 years of age. According to USA lifetime risk for developing S, it accounts for 2.4% in African Americans and 0.85% in white Americans [15,51].

With regards to family history, it was shown that 4–10% of the involved subjects have a relative affected by S [52,53,54].

With respect to ocular involvement, different percentages are published in the literature, ranging from 12% to 70.9%, and the presenting symptom can be localized in the eye in approximately 30–40% of systemic S [55,56,57,58,59,60,61]. These observations further support the hypothesis that environmental, occupational, and para-occupational agents play an important role in the development of S (Table 1).

### 2.2. Etiopathogenesis

The etiopathogenic pathway of S is still unknown. It was recently hypothesized that genetic, epigenetic, and environmental factors interact with an unknown antigenic trigger and induce the following cascade of events: macrophage activation mediated by TLR2, the production of inflammatory cytokines (IL-6, IL-12, IL-18, and TNF α), the activation of CD4+ T cells, and differentiation into TH1 cells with the production of INF- γ and IL-2 and into TH17 cells which secrete IL-17. Subsequent amplification of TH1 immune response due to impaired regulatory T-cell response causes the development of granulomas. The formation of granulomas may occur when the immune system reacts with a protracted T-cell response to the causative agents in the attempt to control a pathogen [62,63,64,65,66,67,68,69].

Many triggers have been recognized to induce S, including bacteria, viruses, dust molds or chemical agents [56]. Genetic factors also play a role in inducing S [69,70,71,72,73,74].

Chaperon found that a single nucleotide polymorphism G16071A in the gene Butyrophilin-like 2 (BTNL-2) seems to be a predisposing cause for S and OS, although no association was found in Caucasian elderly females affected by sarcoid uveitis, mainly carrying the wild-type genotype [74].

Siblings and offspring of subjects suffering from S are five and four times more likely to develop the disease, respectively [75,76,77,78]. Different HLA second class (HLA-DR) alleles have been detected to be S-related [79,80,81,82,83,84,85,86,87,88,89]: HLA DRB1*15 in the Turkish population [90]; HLADRB1*11 in subjects with extra-pulmonary S [91]; HLA DRB1*0401 in patients suffering from OS involvement [92].

To summarize, it appears that S is the result of an exaggerated immune response to an environmental or infective antigen in a genetically predisposed host.

### 2.3. Histopathology

Granulomatous inflammation is a well-defined chronic inflammatory process, in which the activated macrophages play the main role and transform into epithelial cells (which are for this reason known as “epithelioid cells”). Granuloma is a circumscribed area of granulomatous inflammation, consisting of epithelioid cells, lymphocytes, leukocytes and plasma cells. Epithelioid cells sometimes turn into giant cells, consisting of a large amount of cytoplasm containing over twenty small nuclei that are either peripherally located (Langerhans-type giant cells) or distributed in the cytoplasm (foreign body-like giant cells) [93].

Lymphocytes form clusters around epithelioid cells. CD4+ T helper cells contribute in early stages through the release of specific cytokines, which influence the formation and maintenance of the granulomatous lesion. Subsequently, the number of T helper lymphocytes of the granuloma decreases, leaving the place to T CD8+ increase (cytotoxic lymphocytes) which helps in the healing of the granulomatous lesion. Flowcytometry was shown to detect an increased CD8+ ratio in the aqueous humor of subjects suffering from sarcoid uveitis, thus proving that immunophenotyping of localized lymphocytosis in aqueous humor can be a marker for OS [94,95].

Sometimes, a typical wall of collagen fibers at the periphery of the granuloma (ring fibrosis) develops, followed by a hyaline and dense scarring [96,97]. The tendency to fibrosis is typical of some organs (skin and lungs). Fibrosis can sometimes accompany the deposit of substances such as oxalate or calcium carbonate [96,98].

Some inclusion bodies can be found inside granulomas leading to the suspicion of sarcoidosis, even though they are not pathognomonic. These bodies are (a) the Schaumann (conchoidal) bodies, laminated, and birefringent concretions consisting of calcium and proteins usually in the cytoplasm of giant cells [97] and (b) the Hamazaki–Wesenberg bodies which are brownish bodies of lysosomal origin which vary in shape (from oval to fusiform) [98]. 

In addition to the classic non-necrotizing granulomatous form, S can give rise to other histopathological subtypes, namely, necrotizing sarcoid granulomatosis and nodular S.

Necrotizing sarcoid granulomatosis is a rare, poorly recognized histopathological condition (a form of S or maybe a separate entity) affecting the lungs, associated with lymphocytic vasculitis [99].

Nodular S is a histological variant characterized by non-necrotizing confluent granulomas accompanied by diffuse fibrosis [93].

Thanks to these histopathological findings, a correct diagnosis of S is achieved through the examination of the affected tissue biopsy, usually showing typical non-caseating and non-necrotizing granulomas.

It must be taken into consideration that histopathology is not pathognomonic because similar findings are also detectable in other granulomatous disorders [100].

According to the literature, a complete diagnostic work-up, including medical history, clinical, radiological, and histopathological findings, is required to obtain a correct diagnosis and to differentiate S from other granulomatous-related disorders.

### 2.4. Systemic Sarcoidosis

Although over 50% of S-affected subjects experience a spontaneous remission of the disease, a variable percentage of them, ranging from 10% to 30%, suffer from a chronic and progressive course. Subjects with black ethnic backgrounds undergo a more symptomatic and severe disease course compared to those of white ethnicity. Staging systems were proposed, and chest radiographic stages provide useful information and prognostic values, although no biological marker in BAL or serum has been available until now to characterize other systemic localizations without pulmonary involvement [101,102,103,104,105,106].

In the USA, lifetime mortality is lower than 5% and is frequently due to lung or heart failure [107].

Several patients are asymptomatic at onset (more than one-third) or may complain of weight loss, fever, fatigue, shortness of breath, cough, chest pain, polyarthritis, erythema nodosum [60].

Heerfordt–Waldenström syndrome, also called uveoparotid fever, can be a presentation of S with uveitis, parotitis, fever, and sometimes facial nerve palsy [108,109].

Thanks to recent epidemiological studies, the predominantly involved organs in S were identified in order of frequency as lymph nodes (90–95%), lungs (>90%), liver/spleen (50–80%), skin (25%), eye (20–50%), and CNS (10%). Clinically isolated uveitis remains a strictly ocular condition in a lot of cases [60,110].

### 2.5. Ocular Manifestations

Granulomatous inflammation might affect any part of the eye, its adnexa and the orbital region.

Eyelid involvement is rare and has been described in individual case reports. The main lacrimal gland is most commonly affected and keratoconjunctivitis sicca (KCS) secondary to lacrimal gland inflammation is a frequent association. The involvement of lacrimal glands is the most frequent form of orbital S. It mimics orbital masses and reflects enlargement of involved lacrimal glands because they have native lymphocytes. It should be considered in the differential diagnosis of other systemic disorders, such as lymphoproliferative diseases (e.g., lymphoma), and inflammatory/infectious diseases (e.g., Sjogren syndrome, granulomatosis with polyangiitis or tuberculosis) [1,3,61,111]. Symptoms include itching, burning and foreign body sensation [1,3,4]. Ptosis, proptosis, strabismus and palpable masses are typical signs of involvement of orbital tissues and extraocular muscles [111]. Nasolacrimal duct obstruction is a sign that the drainage system is affected [112]. These kinds of manifestations need to be differentiated from other orbital inflammatory conditions (e.g., thyroid ophthalmopathy) [113].

Neuro-sarcoidosis is an uncommon but potentially serious manifestation of S, sometimes it is called “the great imitator” because it mimics signs and symptoms of many diseases. Cranial nerve involvement can occur due to direct infiltration by sarcoid tissue or compression from space-occupying lesions. If intracranial inflammatory lesions involve the visual system, they may lead to an abnormal pupillary response, visual field abnormalities, or decreased visual acuity [114]. Optic disc involvement is a less common event and it is limited only to case reports [100]. Papillitis, papilledema, granuloma of the optic nerve [115], optic nerve compression, and optic disc atrophy are the main signs of optic nerve involvement [116]. Papillitis has a typical presentation with optic disc edema and severe visual loss, suggesting a severe outcome. Sarcoid involvement of the optic nerve is easy to diagnose if systemic involvement is present, while it is quite difficult to differentiate sarcoidosic papillitis from other causes of optic nerve involvement if a diagnosis of the systemic disease is not present [100]. Relapses did not influence outcome; a more widespread sarcoid neurological involvement (e.g., meningeal) papillitis is typically progressive with poor prognosis that depends on timing of diagnosis and treatment [114]. MRI with and without contrast is the imaging modality of choice. Gadolinium enhancement is a marker of disease activity and it is a biomarker for response to therapy. Papillitis is seen on MRI as abnormalities restricted to the optic nerve and its sheath [117,118,119,120]. In our experience, optic disc involvement is typically present in patients affected by bilateral OS chorioretinal involvement. Two of our patients presented with unilateral papillitis at onset and, unfortunately, they had a progression to optic disc atrophy because they presented when the optic nerve damage was already advanced.

Conjunctival nodules, acute and chronic conjunctivitis are typical signs of conjunctival involvement [120].

**Figure 1:**
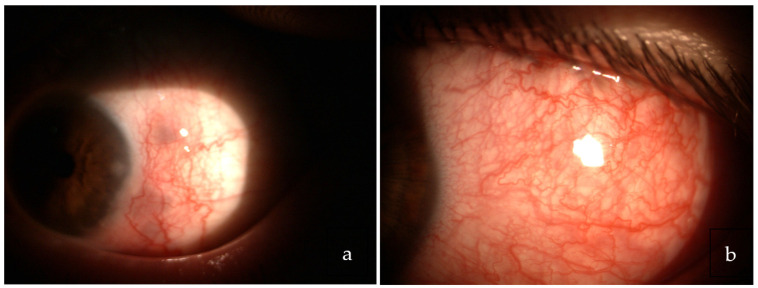
Nodular (**a**) and diffuse (**b**) scleritis in biopsy proven ocular sarcoidosis.

Scleritis related to S is sometimes present, mainly in elderly females, and it can manifest as either non-necrotizing nodular or diffuse anterior or posterior scleritis [121,122] (Figure 1).

Calcific band keratopathy due to subepithelial deposition of calcium, punctate keratitis secondary to keratoconjunctivitis sicca (KCS), interstitial keratitis, and peripheral ulcerative keratitis (PUC) are rarely reported as signs of corneal involvement [1,3,61,123].

The most common ocular manifestation is uveitis, reported in 30–70% of S cases [123]. Bilateral chronic uveitis is present in three-quarters of cases. According to SUN criteria, uveitis is classified in four anatomical forms: anterior, intermediate, posterior, and panuveitis [2].

**Figure 2:**
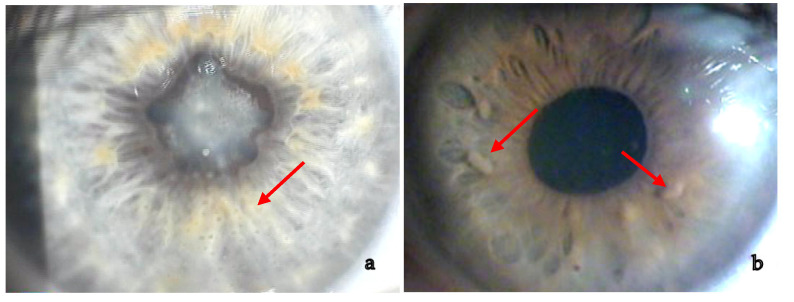
(**a**) Granulomatous anterior uveitis with large keratic endothelial precipitates mainly spread in the inferior part of the cornea. (**b**) Busacca iris nodules.

Anterior granulomatous uveitis with anterior or posterior synechiae (20–23%) and keratic precipitates in the lower half of the cornea (if white and not pigmented this represents active inflammation) is the most frequent presentation (60–80%). Nodules on the pupillary margin (Koeppe) or in the stroma (Busacca) are present when uveitis is active, their regression is a sign of therapeutical efficacy [1,3,6,60,123] (Figure 2).

Intermediate uveitis presents with floaters and blurry vision, dense vitreous opacities, aggregates of inflammatory cells (snowballs) or accumulation of white fibrous exudates (snowbanks) at the level of pars plana [1,3,41,59].

**Figure 3:**
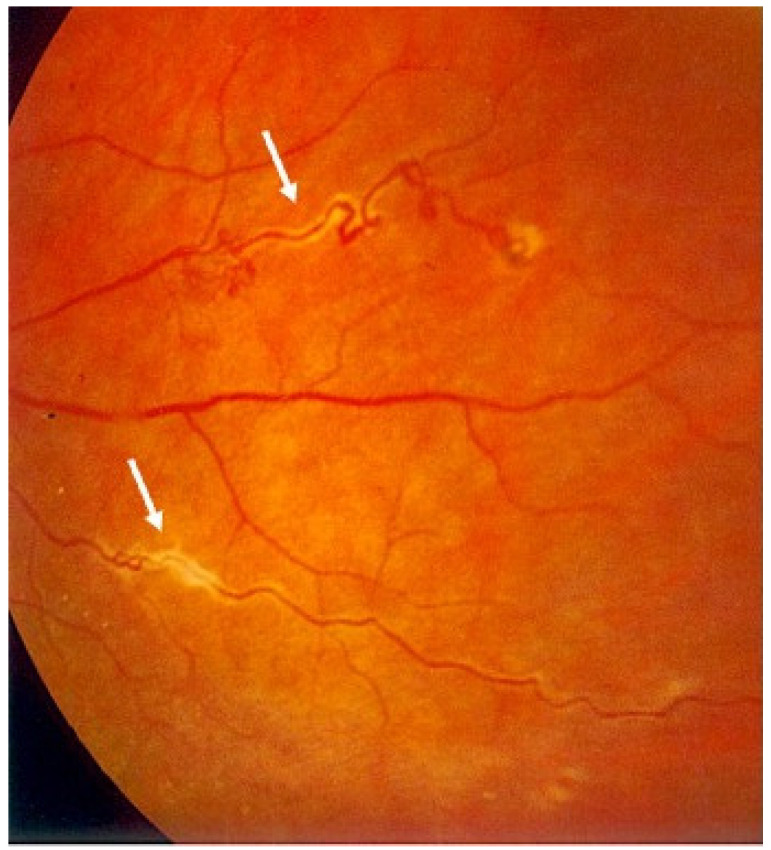
Typical sectorial vascular sheathing resembling “candle wax drippings”.

Posterior involvement is usually bilateral but asymmetric. Choroidal granulomas vary widely in number and size and can lead to visual impairment [124]. CNV may develop at the edge of these lesions. Exudative retinal detachment can occur when choroidal granulomas are very large. Periphlebitis, vascular sheathing and “candle wax drippings” (scattered whitish-yellow perivascular retinal exudates along the retinal veins) are common findings (Figure 3). Retinal vasculitis is infrequently occlusive, but retinal vein and arterial occlusion were reported in the literature [1,125,126,127,128,129,130,131,132]. Occlusive retinal vascular diseases such as branch or central retinal vein occlusion have been reported. According to our experience, retinal venous occlusions and neovascularization are quite rare and may be explained by direct microvascular ischemia rather than inflammation or granulomatous infiltration, or it may be bound to perivascular proliferative changes (vascular sheathing) compressing the vessels and leading to luminal occlusion [1].

### 2.6. Ocular Complications

Complications of chronic intra-ocular inflammation include, in order of frequency: cataract, CME, and glaucoma, which can all lead to reversible or permanent vision loss. Cataract is very frequent, either due to the chronic use of local, intraocular or systemic steroids, or to the presence of persisting inflammation [1,3,124,131].

**Figure 4:**
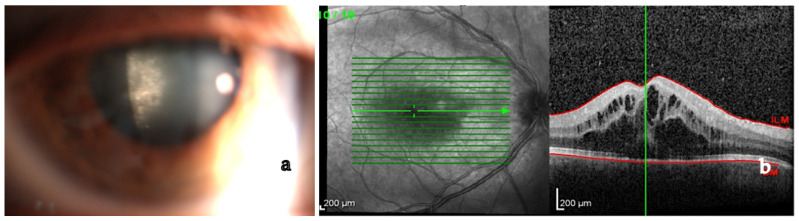
Ocular sarcoidosis complications: subcapsular cataract (**a**), acute CME (**b**).

CME is frequently associated with retinal vasculitis or severe active chorioretinal inflammation. Epiretinal membranes or macular pucker are frequently found, especially when CME regresses [1,3,4,122] (Figure 4).

Sarcoid uveitis can lead to increased IOP and, consequently, glaucoma primarily due to steroid therapy, edema, debris or inflammatory cells that cause trabecular meshwork obstruction, alone or in combination. Granulomatous orbital masses can give rise to high IOP too, as a consequence of compression.

**Figure 5:**
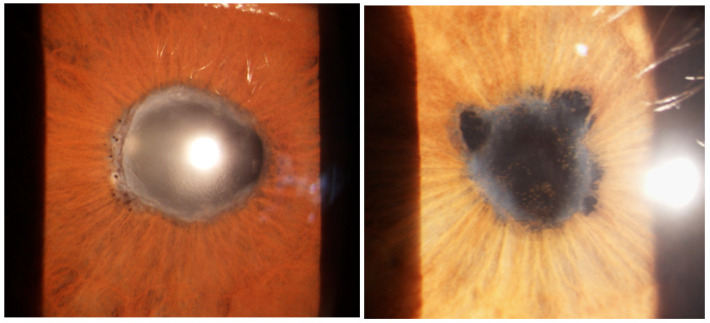
Different presentations of 360° posterior synechiae in OS.

Acute angle-closure glaucoma can be caused by 360° peripheral anterior or posterior synechiae [1,4,61,133,134] (Figure 5).

Gonioscopic findings show trabecular meshwork deposits, nodules, and tent-shaped anterior synechiae in several patients [1,135].

Sarcoid glaucoma is very difficult to treat, and it is one of the leading causes of blindness in these patients owing to its progression despite medical and surgical treatment. Although trabeculectomy associated with the application of Mitomycin-C is a valid surgical choice to treat refractory glaucoma, there is a high rate of failure bound to the high tendency to fibrosis, frequently requiring glaucoma drainage devices as an alternative surgical option [3,135].

### 2.7. Differential Diagnosis

OS should be included in the differential diagnosis of any uveitic onset. Sarcoid uveitis needs to be differentiated from other causes of granulomatous inflammation. Infectious diseases (namely, tuberculosis (TB), syphilis, Lyme disease, different Herpes viruses, toxoplasmosis, and leprosy), autoimmune diseases (Vogt–Koyanagi–Harada disease, systemic vasculitis, ankylosing spondylitis, inflammatory bowel diseases, systemic lupus erithematosus, Behçet’s disease, multiple sclerosis), and other diseases confined to the eye (e.g., birdshot chorioretinitis) should be ruled out. Other diseases to consider in the differential diagnosis of granulomatous uveitis include lens-induced uveitis, lymphoma, histiocytosis X, and neoplastic or para-neoplastic disorders [1,3,4,5,135].

### 2.8. Prognosis

Although complications are very frequent, OS is usually associated with a favorable outcome and a permanent visual impairment is quite rare [9].

A study including 83 patients reported a full recovery of vision in 60% of cases and none developed blindness. Sometimes, severe visual impairment (BCVA inferior to 0.1 in at least one eye) occurs in 2–10% of sarcoid uveitic patients [5,9,22,94]. Uni- or bi-laterality, chronicity and severity affect the visual prognosis of subjects suffering from OS. Those of female gender, elderly age, black ethnicity, or with persistent ocular inflammation (especially posterior) and complications are more prone to a worse visual prognosis [60,61,96,124,135]. Edelsten, in a prognostic study, determined that visual loss was mainly bound to glaucoma and CME [134], while Dana showed that approximately 90% of patients suffered from chronic uveitis [78,132]. A good prognosis generally depends on an early diagnosis and prompt treatment. Severe posterior uveitis and optic nerve involvement are sight-threatening conditions and, together with neurosarcoidosis, they are absolute criteria for systemic steroid treatment, although randomized studies concerning the efficacy of this treatment are not available at the moment. In steroid-refractory patients, immunosuppressants can be added [1,119].

### 2.9. Systemic Diagnostic Procedures

Diagnosing S remains extremely challenging. Systemic S, although involving any organ of the body, usually affects, in order of frequency, the lungs, mediastinal lymph nodes, heart, liver, spleen, eye, and brain [136].

A careful assessment of the clinical history and medical examination are usually useful tools for achieving a correct diagnosis, although biotical histological confirmation is always required [1,3,4,59,96,123].

Tissue biopsy (mainly sampled from lungs, lymph nodes, skin, liver, orbital, lacrimal gland, and conjunctival tissues) is the diagnostic gold standard [20,52,136,137].

Following the IWOS revised guidelines [7], patients with typical findings who are not eligible for biopsy need to undergo specific laboratory tests, chest HRCT and, if available, ^18^F-FDG-PET [137].

So far, reliable biomarkers of S are not available in routine clinical practice.

Laboratory diagnostic tests include: Quantiferon-TB gold and VDRL-TPHA-RPR to rule out TB and syphilis, respectively, since they have a similar presentation to S. Serum ACE and lysozyme levels can be high in these patients, since they are markers of granulomatous inflammation. ACE levels lack in specificity and have a limited clinical usefulness, because they are usually high in unaffected children, low in subjects undergoing steroid treatment and altered in patients under ACE inhibitors [1,3,10,60,96,138,139]. Lymphopenia was also recognized by Jones as an independent predictor of S in subjects with uveitis [140]. Increased values of ACE, lysozyme, ß-2 microglobulin indicate an active disease [141,142,143]. Increased production of vitamin D from epithelioid cells causes hypercalciuria and less frequently hypercalcemia [144]. More recently, the soluble interleukin-2 receptor (sIL-2R) produced by T helper cells and alveolar macrophages was introduced in the laboratory examination panel of subjects suffering from S. This test is used as a biomarker for disease severity in S, for distinguishing patients from healthy controls and active from inactive disease, as well as for assessing treatment success. sIL-2R also correlates with other biomarkers, including ACE, and with lung function tests and nuclear imaging studies [143]. Other useful tests are the skin pathergy test and B lymphocyte polyclonal activation [15,16].

A lot of S-affected patients suffer from lung involvement; chest radiography is the first useful step to detect pulmonary involvement. The radiographic staging system can be useful to evaluate prognosis [103,104,105,106].

In cases of negative chest radiography, HRCT imaging detects parenchymal or hilar alterations; it guides biopsy, although it is burdened by the exposure to high levels of radiation [144,145].

**Figure 6:**
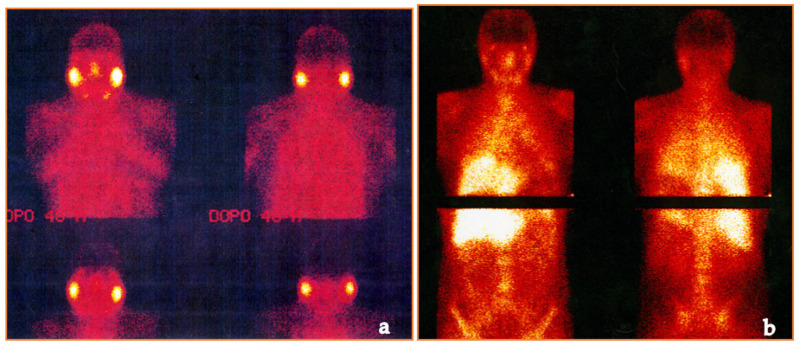
^67^Gallium total body scintigraphy showing increased uptake at salivary glands (**a**) and liver (**b**).

For over 30 years, ^67^Gallium citrate (^67^Ga) scintigraphy scans were used to detect active diseases such as lymphoma and S [146,147]. In recent years, imaging has upgraded with the use of single-photon emission computed tomography (SPECT) and SPECT-CT. A review of Israel and colleagues showed that these radio-diagnostic tools are useful in patients affected by uveitis and liver granulomas with a negative or equivocal chest radiography. The authors concluded that these investigations may have an important diagnostic role in asymptomatic patients [148,149,150] (Figure 6).

A more recent diagnostic technique, ^18^F-FDG PET/CT, enables us to visualize areas of active tissue inflammation and may correlate them with disease activity. However, other diseases can mimic the same “false-positive” result (e.g., lymphoma) [151,152].

BAL may help in S diagnosis: lymphocytosis higher than 15% and CD4/CD8 T-lymphocyte ratio greater than 3.5 can support S diagnosis [52,153,154,155,156].

To assess which clinical and laboratory tests can lead to a correct diagnosis of S, the first international criteria were published in 2009, following the IWOS criteria [6]. These criteria were then updated and published in 2019 [7]. Furthermore, the SUN recently published other criteria [9].

### 2.10. Ocular Diagnostic Procedures

**Figure 7:**
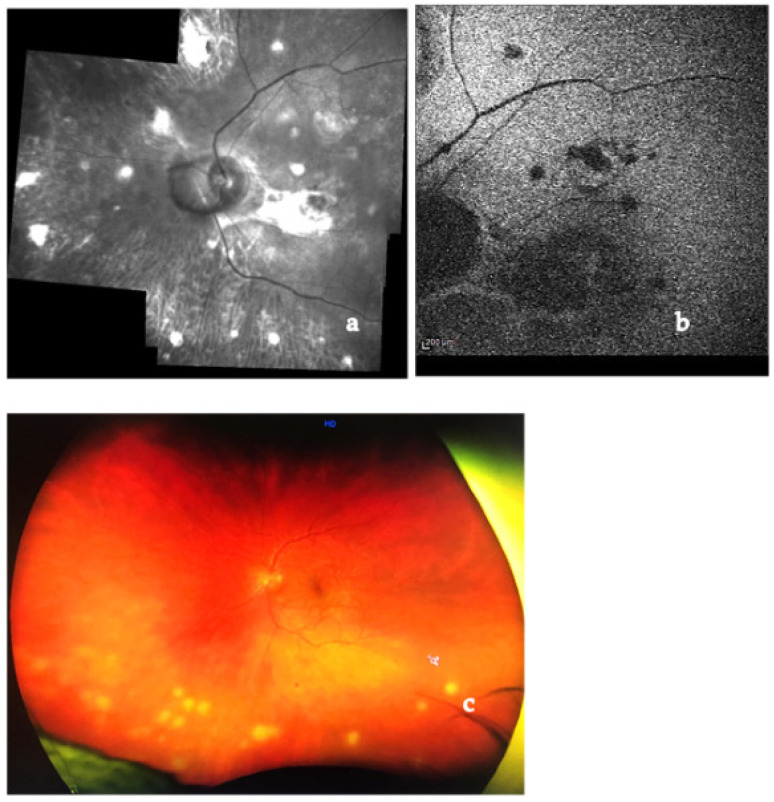
Red-free fundus photography (**a**), BAF (**b**) and UWF (**c**) pictures of a patient affected by retino-choroidal involvement of OS, showing round scattered choroidal granulomas.

Diagnostic and angiographic imaging techniques are useful tools in achieving a correct diagnosis. Multimodal imaging is a new way to detect retino-choroidal pathologies. It consists of the association of different imaging techniques: color fundus photography, IR and red-free fundus photography, BAF, FA, ICGA, OCT, and OCT angiography (OCTA) [157,158,159]. Recently introduced ultra-wide-field (UWF) technology allows us to see images of the chorio-retinal periphery (up to 200 degrees in a single image) (Figure 7). Enhanced depth imaging-OCT (EDI-OCT) allows to see the choroidal layers [160,161].

IR and BAF are useful in S because they show characteristic granulomas and can be used to monitor their evolution in acute or chronic forms over time [157,158,159,160].

**Figure 8:**
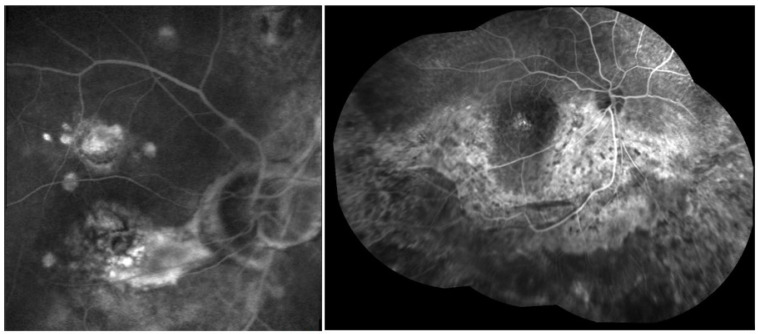
Late phase of FA showing granulomas and CME.

FA in cases of posterior sarcoid uveitis can show:

(1) diffuse or segmental vascular leakage (peri-phlebitis) caused by retinal vasculitis, with vascular sheathing, situated mainly at the middle and extreme periphery;
(2) macroaneurysms (frequently present in elderly females);
(3) areas of vascular non-perfusion or occlusion predominantly involving veins, sometimes mimicking neo-vascularization;
(4) papillitis (usually bilateral and asymmetrical);
(5) acute or chronic CME [161,162,163,164,165] (Figure 8).

Choroidal granulomas are typically scattered and present early hypofluorescence and late hyperfluorescence.

UWF FA is useful to identify peripheral vascular lesions (vasculitis), to detect granulomas and, compared to traditional angiographic techniques, can better show retinal vascular leakage both at posterior pole and the retinal periphery [166,167,168].

**Figure 9:**
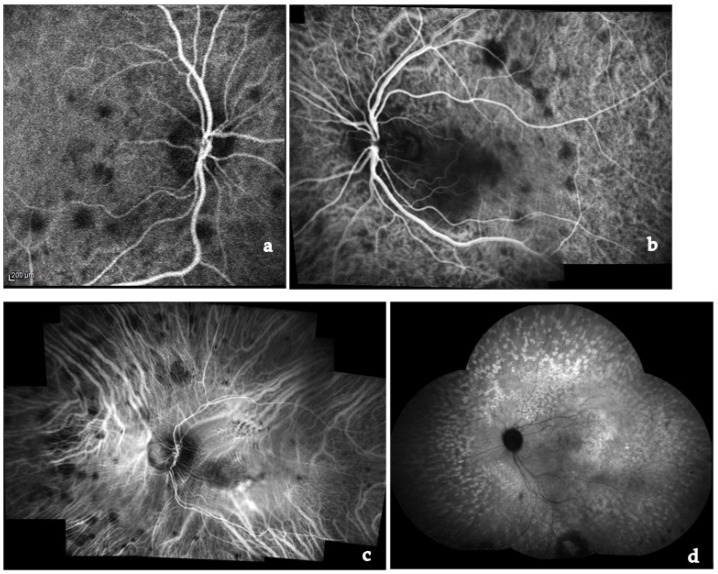
Early phase of ICGA showing multiple hypofluorescent active choroidal granulomas (**a**,**b**); Late phase of ICGA with multiple granulomas evident in the whole retina (**c**,**d**).

ICGA represents the specific diagnostic imaging technique to detect choroidal pathological alterations, mainly granulomas. They appear as dark round formations, irregularly distributed, hypofluorescent in the early and intermediate phases of ICGA [165,169,170,171] (Figure 9). ICGA also detects chorioretinal alterations in subclinical disease and in cases of severe vitreitis with media opacities. Herbort described four different ICGA patterns in choroidal S [170,171].

OCT combined with EDI and combined depth imaging (CDI) and enhanced vitreous imaging (EVI) allows us to grade the intensity of vitreitis, as well as monitor and follow-up CME and subretinal fluid [172,173,174,175,176,177,178,179,180,181,182,183,184,185].

**Figure 10:**
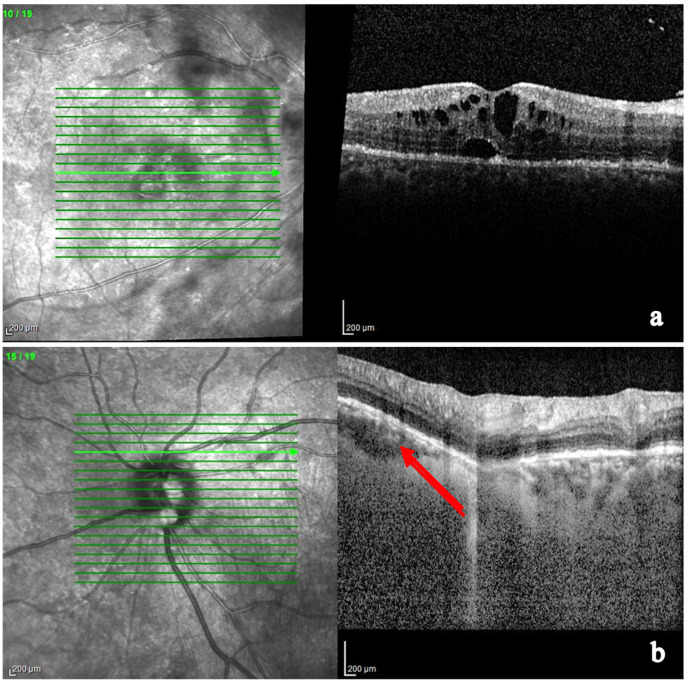
OCT showing chronic CME and a foveal serous detachment (**a**); OCT EDI showing deep choroid and a choroidal granuloma of the optic nerve head (arrow) (**b**).

EDI-OCT detects choroidal granulomas by visualizing choroidal and inner sclera. Granulomas are seen as homogeneous, hyporeflective lesions with well-defined edges in a relatively thinner choriocapillary (thanks to their higher density being non-caseating in nature). Choroidal thickening is seen in posterior scleritis together with a serous retinal detachment [176,177,178,179,180,181,182,183] (Figure 10).

Parrulli demonstrated that both EDI-OCT and ICGA are useful in evaluating choroidal granulomatous lesions. The former enables the visualization of structural changes in choroidal granulomas thickness, monitoring treatment, while the latter can be useful to detect new lesions and the extent of involvement, but not in distinguishing the thickness of the lesions [169]. Therefore, these two investigations are complementary.

Agarwal et al. studied the differences in multimodal imaging between TB and sarcoid granulomas. They showed that, in comparison with S, tubercular granulomas are mainly full-thickness, solitary, lobulated, in the perivascular area. Furthermore, they are larger and vascularized, while sarcoid granulomas are multiple and usually associated with retinal vasculitis and papillitis [180].

It may be very challenging to discriminate between sarcoid choroidal granulomas and amelanotic melanoma or endogenous endophthalmitis. However, well-defined borders are typical of sarcoid granulomas, while melanoma presents homogeneous optical reflectivity, subretinal deposits, and fluid [185].

Swept-source OCT can show a better imaging of deep and peripheral granulomas compared to SD-OCT [184].

**Figure 11:**
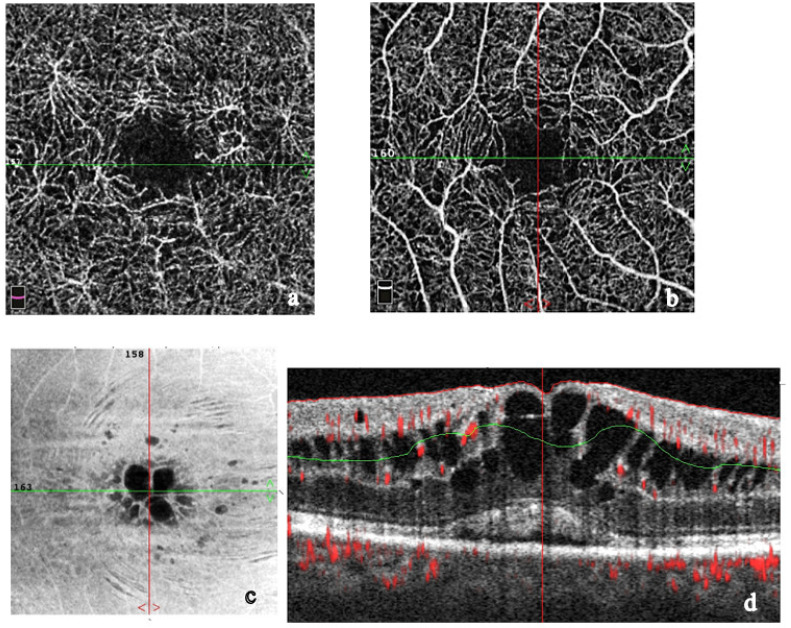
Angio-OCT demonstrating a disruption in the superficial capillary plexus and enlargement of the foveal avascular zone (FAZ) (**a**), with an improvement after three months of systemic therapy (**b**). Angio-OCT showing chronic inflammatory CME (**c**,**d**), especially at the level of the deep capillary plexus (**c**).

OCTA imaging of choroidal granulomas shows dark spots or defects in vascular architecture. It can give a complete histopathological picture of sarcoid lesions. With “en face” imaging, OCTA shows choroidal flow voids in areas which correspond to loss or displacement of choriocapillaris vessels due to granulomas [179,183,184,185] (Figure 11). OCTA imaging is powerful; however, it cannot provide a complete histopathological picture.

Cerquaglia et al. reported OCTA findings in eyes affected by OS and noticed that the superficial capillary plexus (SCP) and the deep capillary plexus (DCP) manifest a different involvement. DCP is more severely compromised with disorganization of the capillary bed, hypoperfused/not perfused areas and cystoid spaces [185].

### 2.11. Medical Treatment

No specific therapy is currently available for OS, but there are different unspecific treatment options to manage the inflammation before the affected eyes develop permanent damage [137]. Therapeutic algorithms for bilateral complicated uveitis have been previously suggested [186,187,188,189,190,191,192,193,194,195,196,197,198].

Following the IWOS recommendations for the management of OS, steroids are the first-line therapy. In anterior uveitis, local steroids should be administered first (prednisolone eye drops up to six times a day). The second-line treatments for moderate to severe anterior uveitis are subconjunctival dexamethasone injections or periocular triamcinolone injections. Eventually, systemic steroids should be considered. For intermediate uveitis, local steroids are the first-line treatment, while systemic immunosuppression is considered second-line for active unilateral or bilateral intermediate uveitis. In case of posterior uveitis, systemic corticosteroids are first-line therapy, alone or in combination with immunosuppressants. Biologic agents should be taken into consideration as an add-on treatment in posterior uveitis [7,199].

Local, peribulbar, sub-tenon, intravitreal and systemic steroids are the mainstay of treatment to prevent complications and treat intraocular inflammation.

Systemic corticosteroids (CS) are introduced when local CS are ineffective, as well as in cases of either bilateral or posterior involvement [188,189].

The initial dose of systemic steroids (prednisone) is 0.5–1 mg/kg/day to a maximum of 80 mg a day, for a mean duration of 2–4 weeks with a low-tapering in 3–6 months.

When the disease is uncontrolled with systemic CS or if high doses of them are needed to control inflammation or in case of refractory disease, additional systemic immunosuppressive drugs and sometimes biologic agents need to be introduced into the therapeutic protocol. Immunosuppressive drugs to be considered are Methotrexate, Azathioprine, Mycophenolate Mofetil, and Cyclosporine A or Tacrolimus.

Some authors recommend treatment of patients affected by sight–threatening conditions (e.g., optic neuropathy) with a combination of high-dosage CS and immunosuppressants right from the onset of symptoms [9,120,137]. In selected cases of aggressive disease, IV-pulsed corticosteroids have to be considered to induce remission of disease.

Biological agents for the treatment of S uveitis are a newly introduced therapy (Table 2). No clinical randomized trial concerning their use in this field is available, although in cases of refractory posterior bilateral involvement they were useful to treat more than 50% of the eyes, even if they needed prosecution of biologic therapy to prevent relapses. They are to be used as a secondary- or tertiary-line treatment because of the lack of clinical data and the report from some studies of a sarcoidosis-like condition as a side-effect of these drugs [1,3,59,60,61,96,102,123,190,191,192,193,194,195,196,197,198,199] The most frequently used is Adalimumab because it is available and on label in several countries [200,201,202].

Unfortunately, there is no specific therapy for OS and the treatment progresses following a stepladder approach. Therefore, standardized guidelines based on randomized clinical trials are advisable.

### 2.12. Surgical and Parasurgical Treatment

Ocular surgery can exacerbate S inflammation. Therefore, mini-invasive surgery is to be preferred. In order to perform any ocular surgical procedure, the eye needs to be clear of inflammation for at least three months. Cataract surgery is frequently performed in these eyes, but CME is a recurrent complication following this surgery [200,201,202].

Glaucoma filtering surgery, although sometimes unsuccessful, is needed in case of low response to local treatment [203,204,205,206,207,208].

Inflammatory CNV can be managed either with systemic CS, immunosuppressants, biological agents or anti-VEGF intravitreal injections [209,210,211,212,213,214].

Refractory CME can be managed with long-lasting intravitreal steroids.

Vitrectomy is performed not only to treat retinal detachment or macular pucker, but also in order to remove dense vitreous opacities and debris [215,216,217].

## 3. Personal Experience

Our study is based on a review of the recent literature and on the experience of our referral center. This retrospective study was conducted at the north-western Italian tertiary referral uveitis center of Rapallo (Genova, Italy). Data were collected from the electronic medical–ophthalmological records of patients in accordance with the principles of the Declaration of Helsinki. This research was approved by an internal medical committee of the same institution and informed consent was obtained from all patients, after a full and detailed explanation of the study was provided.

235 subjects met the inclusion criteria of “definite” biopsy-proven or “presumed” OS (following the IWOS updated guidelines) and were retrospectively analyzed [7].

All subjects underwent systemic physical examination, immunogical/internistic counselling, and follow-up by the same immunological staff. Systemic involvement, if present, was demonstrated by typical findings obtained by the combination of HRCT-guided pulmonary or lymph node or skin biopsy, total body ^18^F-FDG-PET, BAL examination, chest X-ray and/or HRCT and specific laboratory analysis.

All patients underwent a full ophthalmologic examination, including best corrected visual acuity (BCVA) using early treatment diabetic retinopathy study (ETDRS) charts and intraocular pressure (IOP) measurement with applanation tonometry, as well as anterior segment slit lamp biomicroscopy, indirect fundus ophthalmoscopy, color anterior segment and fundus photography. Infrared imaging (IR), blue auto-fluorescence (BAF), optical coherence tomography (OCT), fluorescein angiography (FA) and indocyanine green angiography (ICGA) were performed using Heidelberg Spectralis (Heidelberg Engineering, Heidelberg Germany).

The purpose of our work is to describe OS manifestations, together with clinical and therapeutical experience of our Uveitis Referral center in a ten-year retrospective evaluation from January 2012 to January 2022.

Among the enrolled subjects, 158 were females (67.2%) and 77 were males (32.8%) and their age ranged from 35–81 years old with a mean of 52 (+4.7) years.

Of these, 172 patients (73.2%) suffered from biopsy-proven (“definite”) OS and only 63 patients (26.8%) from a “presumed” form (typical ocular characteristics of S without any systemic involvement during the course of the ocular disease).

78.3% of patients underwent tissue biopsy for a total of 184 biopsies. Among them, 122 (66.3%) were performed on enlarged lymph nodes, 31 (16.8%) on lungs, 15 (8.1%) on livers, 13 (7%) on skin granulomas, and 3 (1.6%) on conjunctival mucosa.

If the results of the biopsy were unconclusive, a total body ^18^F-FDG PET was scheduled, and a new thoracic/lung HRCT was performed before undergoing another biopsy.

**Figure 12:**
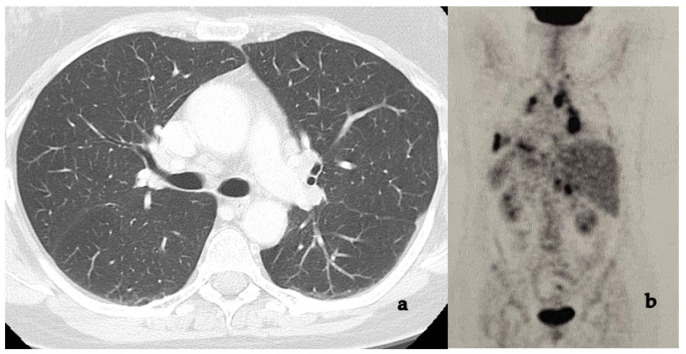
HRCT (enlargement of hilar lymph nodes) (**a**) and total body 18F-FDG PET (multiple areas of increased uptake corresponding to regions of active disease) (**b**).

Clinical data were based on the IWOS criteria and included intraocular clinical signs and systemic investigations [7] (Figure 12).

Data collection from laboratory testing was implemented with the following additional examinations: syphilis serology (VDRL-TPHA), liver enzyme tests (alkaline phosphatase, aspartate transaminase, alanine transaminase, gamma-glutamyl transferase), calcemia, and calciuria.

Results of our retrospective analysis are shown in the following tables (Table 3, Table 4, Table 5, Table 6, Table 7 and Table 8).

Concerning treatment in out cohort, some patients were not responsive to immunosuppressive treatment in association with low dose systemic CS after 6 months or they manifested relevant side effects that forced discontinuation of therapy, therefore they shifted to biologic agents. In particular, 1 in 12 patients treated with Azathioprine did not respond to treatment, nor did 5 in 29 treated with Methotrexate, nor 4 in 43 treated with Micophenolate Mofetil. Immunosuppressive treatment is usually discontinued after about 12–24 months thanks to disease remission. Due to severe relapses, 2 patients on Azathioprine, 2 on Methotrexate, and 10 on Micophenolate Mofetil are still undergoing treatment after more than 5 years. These are the three immunosuppressants that we used to treat our patients, in accordance with the suggestions of the immunologist.

Moreover, a patient who received Adalimumab needed to stop biologic treatment due to the development of anti-Adalimumab antibodies. Half of the patients treated with Adalimumab (5 in 10 patients) are still receiving biologic therapy after 5 years because of CME relapses upon discontinuation of the drug. Patients treated with Interferon α-2a and Rituximab were able to successfully stop therapy after 24 months with no relapses.

## 4. Discussion

### 4.1. Systemic Involvement

S is a granulomatous disease causing non-caseating granulomatous inflammation in genetically predisposed subjects. The clinical manifestations of S are variable and frequently leading to a late diagnosis and, therefore, chronicity. Any organ of the body may be affected, including the eye, despite the lung being the primary target organ. When the disease is confined to the eye, there is frequently a lag in the diagnosis and it may go unrecognized [1,3,4,51,61,94,122,124,125,133,202].

Our study is focused on a recent literature review associated with 10 years’ experience from our tertiary referral center (Rapallo Hospital, Genova, Italy). We retrospectively analyzed a cohort of 235 subjects suffering from either biopsy-proven OS (172 patients, 73.2%) or from the “presumed” form of OS (63 subjects representing 26.8%). Following the updated IWOS criteria, the former group was classified as “definite” S, while the latter as “presumed” since no biopsy was performed but positive bilateral lymphadenopathy associated with typical ocular and laboratory findings of S were detected [7,9]. A third form of the disease is defined as “probable” by the IWOS criteria, however we decided not to include it in our investigation because of its diagnostic unreliability. Updated IWOS criteria were used as a reference for classifying intra-ocular uveitic sarcoid involvement in patients. According to our experience, these criteria are rather easy to apply for classification purposes.

Along with other authors, we agree that diagnosis of S is relatively simple when systemic symptoms and signs are present. On the other hand, it is rather tricky if S is limited to one organ such as the eye [1,3,59,122,125].

A definite diagnosis is confirmed through tissue biopsy showing non-caseating granulomas. Conversely, caseating granulomas are typical of infectious diseases, namely, TB, or mycoses, they present a necrotic central area and have a “cheese-like” appearance. Noncaseating granulomas are more frequently found in inflammatory diseases such as S and typically do not include necrotic areas. A typical non-caseating granuloma is characterized by a core of macrophages framed by a wall of helper T-cells; macrophage proliferation is induced by the TH_1_ Subtype T-cells [18,52,61].

S can develop regardless of ethnicity or age. African Americans show a higher incidence of the disease, as do Scandinavians as compared to the rest of the Caucasian population [25,26,27,28,29,30,31,32,33]. The great majority of the patients we examined were Caucasians (221 of 235 patients, meaning 94%), given the predominance of Caucasian ethnicity among the resident population. The remaining 14 (6%) subjects were represented by six South Americans, four north Africans, three patients from India, and one Chinese. No African American was evaluated at our center. The high Caucasian prevalence in our investigation gives strength to our data, thanks to the homogeneity of the study group [218,219,220,221,222].

S incidence is estimated to be between 2.3 and 11 per 100,000 individuals/year (Table 9). The expected prevalence varies from 2.17 to 160 per 100,000 individuals. This high variability could be explained by the limited diagnostic tools employed in older case series and by the different ethnicity of each cohort [4,27,28,29,30,31,32,33,34,35,36,37,38,39,40,41,42,43,44,45,46,47,48,49,50,223]. An American five-year study showed that the age-adjusted annual incidence was 10.9 per 100,000 among Caucasian Americans, and 35.5 per 100,000 for African Americans [42]. A study from Beghè et al. observed that the prevalence in the province of Parma over the period 2000–2013 was 49 cases per 100.000 individuals [218,219].

Although epidemiological data about S in our region (Liguria) are missing in the literature, systemic S is frequently found affecting the local population at our tertiary referral center of Rapallo (Genova—Italy). The etiological incidence of sarcoid uveitis accounts for about 8% (personal unpublished data) of the total number of uveitis/year. In our opinion, this high incidence is related to the significant presence of mine workers and to a racial genetic predisposition of mainly Caucasian people living in this area. Caucasian subjects were found to be the most frequently affected by posterior and anterior sarcoid uveitis as shown originally by Rothova and by Dammacco 20 years later, confirming our data [3,57].

S onset is usually in adults between 25 and 40 years of age (70%), although children may also be affected. A second peak of incidence is typical in Caucasian women over 50 years of age [3]. In our center, no affected children were visited, therefore only adults were included in the retrospective analysis. Our study shows that 158 (67.2%) of the studied subjects were females, while males represented only one-third of the cases (32.8%). The mean age at onset of the systemic disease in our cohort was 47 ± 3.6 years; however, the first ocular manifestation occurred at 52 ± 4.7 years. Hence, OS manifested at an older age compared to the systemic disease.

According to some studies, the disease is more frequent in young adults, but the first diagnosis is made later, usually in people with a mean age of approximately 50 years [1,3,13,59,96,223,224,225,226,227], confirming our data. In the ACCESS study (A Case-Control Etiologic Study of Sarcoidosis) the peak age was between 35 and 45 years [14], while in a UK survey it was found to be between 35 and 55 [78]. The review by Valeyre [20] shows that 70% of patients are diagnosed between 25 and 45 years old. It is remarkable to observe that age at diagnosis usually does not coincide with the onset, probably because of the asymptomatic course or the mimicking of symptoms and signs of other diseases, together with the usually accidental discovery of the disease by routine X-ray examination [224]. Musellim et al. [223] identified a higher mean age at diagnosis for female patients compared to males, with a difference of 10 years between the two genders. We found a similar result concerning the age of the studied patients, with a mean age at diagnosis of around 53 years for females and 46 years for males, confirming previous data that a younger age of onset is characteristic of males. In our study, more than one-third of females (57 patients, 36%) were older than 60 years, confirming the results of Scandinavian, Japanese and Spanish studies [45,48,220,221,222,224]. This might be due, in our opinion, to an early asymptomatic disease or to a late diagnosis because of the worsening of symptoms and signs. The Orphanet Reporter Series for Rare Diseases includes sarcoidosis in the registry of rare diseases and reports an estimate of the mean worldwide S prevalence to be 12.5 cases per 100,000 individuals.

The work by Beghè [219], conducted on subjects living in the area of an Italian province (Parma), revealed a higher prevalence of S compared to other studies, estimated at 50 cases per 100,000. This can be interpreted in two different ways: either environmental triggers can elicit the disease onset, or S is much more common than predicted [50,51] due to many asymptomatic forms.

We were not able to assess the prevalence of the disease in our population because of the multi-regional origin of our patients.

Regarding ophthalmological involvement, bilateral involvement was detected along the course of the ocular disease in 393 eyes (85.2% of the studied eyes). Dana showed that bilaterality was the main presentation; 20 years later, our study confirms data in existing literature [225].

Our retrospective analysis showed that systemic S was diagnosed as follows: in 35 patients (14.9%) prior to ocular involvement; in 120 subjects (51%) with simultaneous systemic and ocular presentation; in 52 patients (22.1%) in a subsequent period of 3–19 months. In our cohort, 172 patients (73.2%) had biopsy-proven (“definite”) OS and only 26.8% (63 patients) suffered from a “presumed” form of OS with typical ocular characteristics at onset. Only 28 subjects (11.9%) did not show any systemic involvement during the follow-up period. Previous studies [5,59,60,94,222] showed that over one-third of subjects presented systemic sarcoid associations together with ocular inflammatory manifestations. Uveitic presentation at first diagnosis had a high variability in these works, ranging from 20–30% to 80%. In our study, more than 66% of subjects presented with a previous systemic ocular association, confirming the variability of the symptoms in different centers including diverse populations. This may be also due to the early or late diagnosis and to the availability of specific tests [1,3,5,122]. Biopsy of the involved tissues, if positive, confirms the disease; it is also supported by some specific diagnostic tests that demonstrate systemic involvement such as ACE, lysozyme, and more recently S-IL-2R. Indeed, among laboratory tests, increased ACE levels were identified as a useful parameter of disease activity, but they lack significance in children (because of their increased bone metabolism) and in patients under ACE-inhibitors or anti-hypertensive treatment [226]. Increased ACE levels were only observed in 66 patients (28%) in our study, and this result confirmed the low specificity of ACE testing alone for the diagnosis of S, as already recognized in other studies [10,226].

Lysozyme was tested in one-third of our patients and 39.7% of them showed increased levels, whereas calcemia and calciuria were increased in about half of the examined patients (51.5 and 54%, respectively).

The recent published work of Papasavvas studied 37 patients affected by suspected or proven S, and tested them for ACE, lysozyme, and polyclonal antibody activation. The authors concluded that lysozyme and polyclonal antibody activation are more useful than ACE to support the diagnosis of OS [227,228,229,230,231,232,233,234].

We tested S-IL-2R dosage in only 43 patients due to its recent introduction and it showed increased levels in 83.7%, thus confirming previous data of zur Bonsen [235] concerning the high sensibility of this test (70.6% in their casistic). We believe it might represent a biomarker to detect the early stage of OS and it has been suggested as a diagnostic tool for S [143,228,229,230,231]. These parameters are also related to malignancies (mainly lymphoma) or infections, thus these pathologies must be ruled out in the differential diagnosis [10,12,61].

Most of our patients underwent chest HRCT (215 pts, 91.5%), followed by ^18^F-FDG PET (183 pts, 77.9%), and BAL (107 pts, 45.5%). 60.4% of thoracic HRCT showed hilar and/or mediastinal lymphadenomegaly; 40% showed parenchymal involvement, 13.2% had a combination of the two previous findings; it was unremarkable in 16 patients (6.8%). Our findings are in accordance with the literature [232,233,234]. Among the 183 patients (77.9% of the enrolled subjects) who underwent ^18^F-FDG PET, an active systemic disease was detected in 42.1% of them. Thoracic involvement was present in 34 (14.5%) subjects, with an extrathoracic localization in 11 (4.7%), and a combination of the two in 22.9%.

Dammacco [57] reported that ^18^F-FDG PET/CT showed hypermetabolism in many organs, with primary involvement in the lungs, allowing for the identification of the most accessible biopsy site.

A number of studies investigating the usefulness of ^18^F-FDG PET/CT in suspected OS reported promising results [235,236].

Rahmi confirmed its usefulness in elderly patients affected by uveitis related to S and recommended this diagnostic tool in cases of normal HRCT [237]. Jamilloux [238] recommended it in cases with suspected OS in which prior investigations were non-diagnostic. In contrast, Burger showed in his retrospective study on 29 patients that ^18^F-FDG PET/CT does not give additional benefit over HRCT in uveitis subjects with characteristics related to S [239]. Our study showed a high usefulness of HRCT in diagnosing S systemic involvement, while ^18^F-FDG PET/CT seems to be a very expensive, although very useful, examination, and therefore should be considered as a second instance diagnostic tool.

In our retrospective study, BAL findings were considered positive if alveolar lymphocytosis was superior to 15% and CD4/CD8 value was more than 3.5 [240]. BAL was positive in 72 patients (67.3%), and unspecific in 35 patients (32.7%). Caspers’ retrospective study on 109 suspected OS patients who underwent BAL and chest imaging showed that BAL was positive in 26.6% of patients (86.2% females, mean age 50.8y) with mean alveolar lymphocytosis (aL) at 46.8% and mean alveolar CD4/CD8 = 8.5. BAL (+) patients had 31% of bilateral hilar adenopathy, therefore they concluded that their findings suggest that BAL has a high diagnostic value and can be a useful additional test in cases with normal chest imaging [241]. These results are in accordance with our findings.

**Figure 13:**
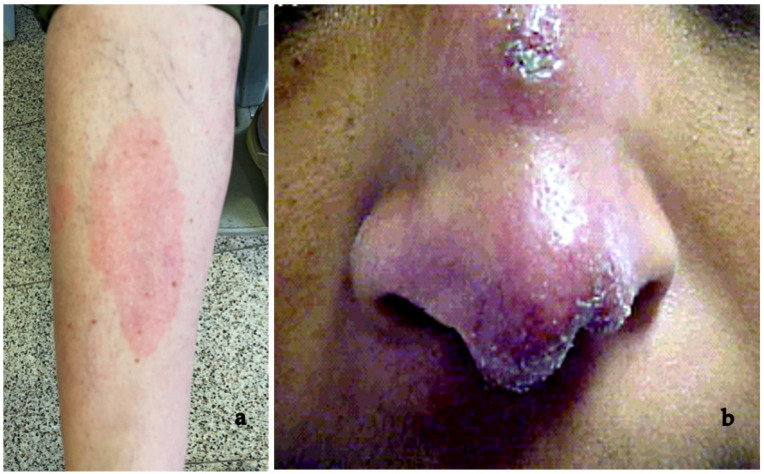
Skin sarcoid involvement. Erythema nodosum (**a**); lupus pernio (**b**).

Dermatologic consultation can also help making a correct diagnosis by detecting S of the skin in the form of nodules, lupus pernio, and erythema nodosum. Dermatological manifestations were present in some patients from our study, confirming previous findings [1,3,61] (Figure 13).

### 4.2. Ocular Involvement

Granulomatous involvement of the orbit, adnexa, lacrimal gland, conjunctiva, and sclera was present in only 21 subjects (8.9%), while uveitis was observed in more than 90%. Vahdani’s thirty-year retrospective study found only 61 patients affected by orbitopathy in this long period of time. Systemic involvement was found in 23 (62%) patients initially presenting with orbital disease [242].

**Figure 14:**
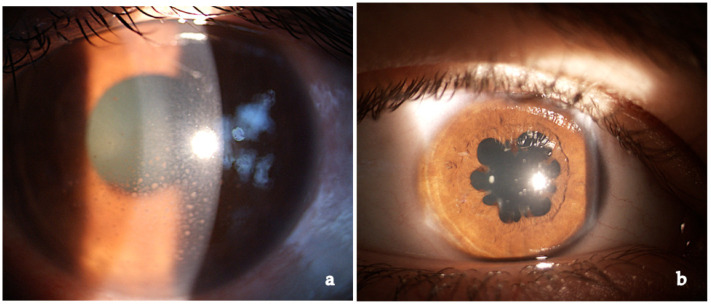
Typical sarcoidosic anterior granulomatous uveitis with mutton-fat keratic precipitates (**a**) and “flower-like” posterior synechiae (**b**).

Anterior acute granulomatous uveitis with mutton-fat keratic precipitates and synechiae, sometimes with iris nodules, was present in more than 50% of our patients (112 subjects—52.3%) at onset (Figure 14). The other uveitic forms, in order of incidence, are posterior uveitis (62–28.9%), intermediate uveitis (22–10.3%) and panuveitis (18–8.4%). Dana found 81% of granulomatous uveitis at onset [225]. In the study by zur Bonsen, anterior uveitis accounted for 31%, while intermediate uveitis for 32.1%, in line with previous observations of a homogeneous distribution in the anatomical settings [228]. A German study reported a 76% of anterior uveitic cases [55].

**Figure 15:**
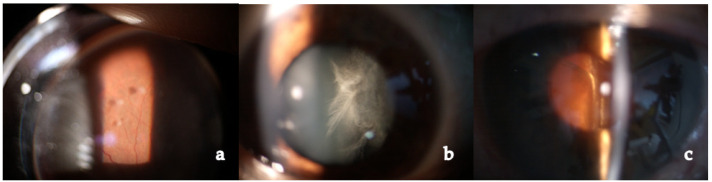
Different aspects of intermediate sarcoid uveitis. Snowballs (**a**); acute inflammatory vitreitis (**b**); chronic vitreitis (**c**).

Intermediate uveitis in the form of strings of pearl-like vitreous opacities with vitreitis and peripheral vasculitis was present in a limited number of our subjects (22 examined patients, 10.3%) (Figure 15).

**Figure 16:**
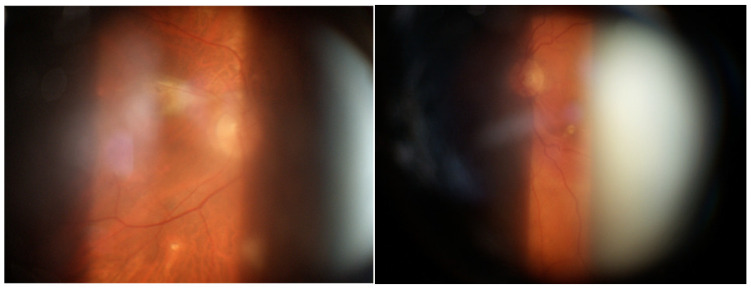
Bilateral chorio-retinal involvement in a patient with OS.

Posterior uveitis was the typical finding in our patients. It is characterized by non-occlusive retinal periphlebitis (especially venous), vascular sheathing, candle-wax drippings (perivenous exudates), multifocal choroiditis (mainly in the periphery), hemorrhagic retinopathy, multiple serous RPE detachments, CME, papillitis, optic nerve granulomas, papilledema, and retinal arteriolitis with macro-aneurisms (mainly in older patients) (Figure 16). It was present in a fairly high percentage of patients, reaching almost one-third of cases, more than previously described by other authors [59,228]. This high incidence may be related, in our opinion, to a diagnostic and/or therapeutic delay in our group of patients and to the chronic evolution of the disease, probably due to a genetic predisposition. A reason for these dissimilarities may be found in the heterogeneity of the populations included in previous studies (i.e., different ethnicity). Moreover, patients affected by severe disease are usually treated at referral centers and, therefore, present different incidence and characteristics as compared to general hospitals.

**Figure 17:**
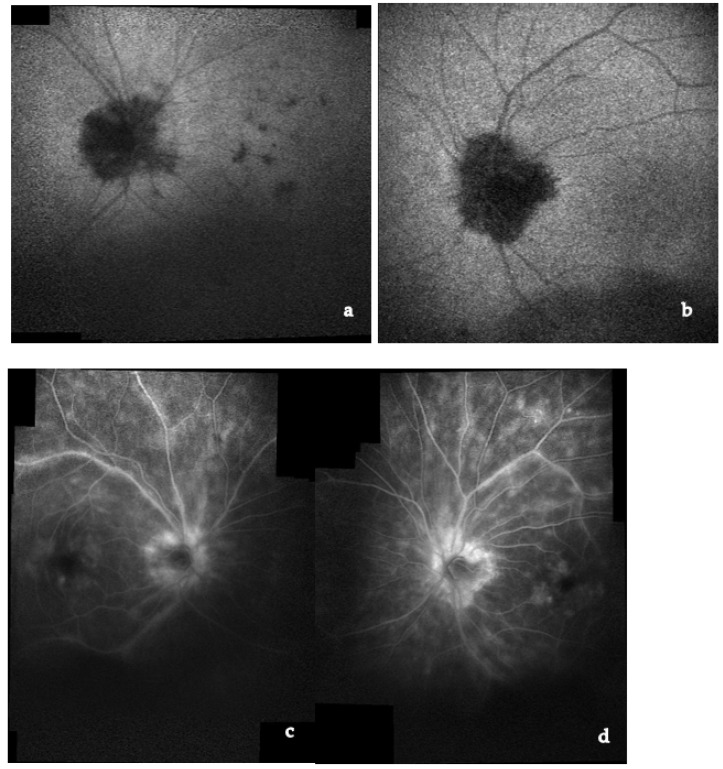
Ocular imaging of a 64-year-old male patient affected by CNS and bilateral optic disc sarcoid inflammation. ICGA late phases (**a**,**b**). FA (**c**,**d**) showing late dye diffusion mainly from the papillary area where granulomas are located; CME and typical segmental vasculitis are also visible.

**Figure 18:**
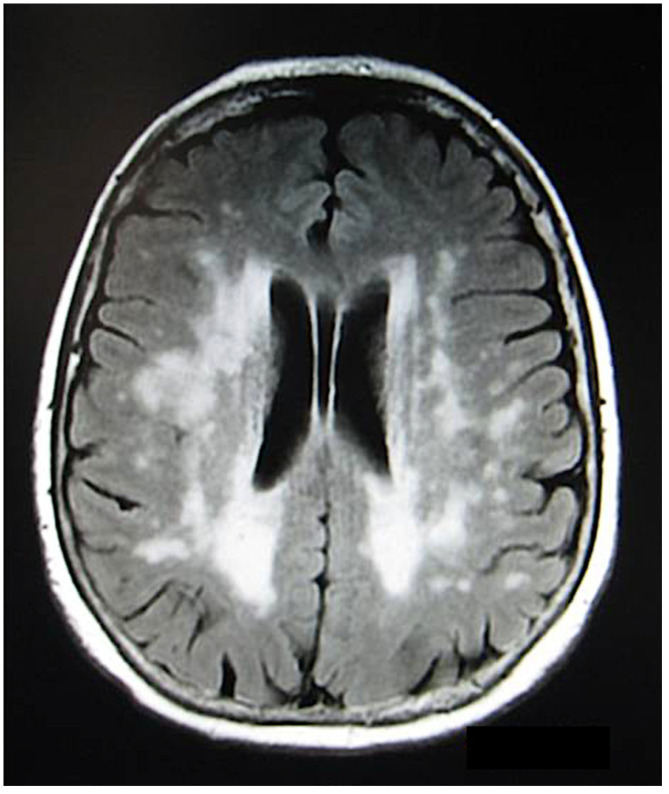
MRI encephalic scan showing typical Gadolinium diffusion due to encephalic vasculitis in patient from Figure 17.

Posterior uveitis related to CNS involvement was present in 14.9% (35 patients) of the cases, probably because of the Caucasian ethnicity of our patients. In our experience, CNS imaging examinations are useful tools to detect CNS involvement associated with posterior uveitis. Clinically significant nervous system involvement (neurosarcoidosis (NS)) occurs in 5–10% of patients with S, but the association of posterior segment and neurological involvement in S has been reported to be as high as 27% [243,244,245] (Figure 17 and Figure 18).

**Figure 19:**
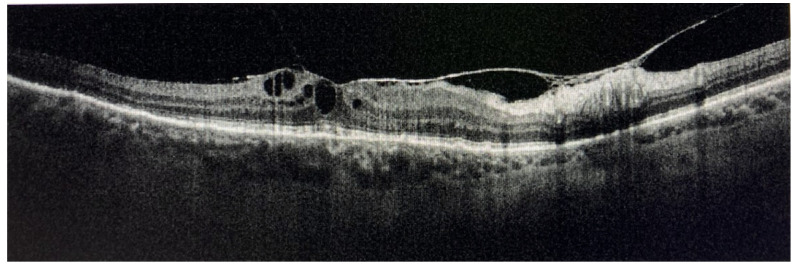
OCT: typical thick vitreoretinal traction in the macular area. CME is also evident.

Vision threatening complications requiring surgical or parasurgical treatment in our group of patients include CME (37.9%) epiretinal membrane (28.9%), secondary glaucoma in more than 10%, and CNV (2.9%) (Figure 19).

Radosavljevic et al. noticed that 20.7% of their patients suffered from glaucoma and Reid showed that 36% of sarcoid uveitis patients suffered from high IOP [49,245].

Among retinal complications of S in our cohort, CME was the most frequent complication (31.9%—147 eyes), mainly affecting eyes with posterior or pan-uveitis, resulting in chronic and untreatable S in 2.9% of eyes [246]. CME complicating unilateral forms of S uveitis was treated with 0.7 mg Dexamethasone intravitreal implant (Ozurdex, Allergan) which was repeated every 4–5 months if CME or posterior inflammation relapsed. Occasionally, parabulbar or subtenon injections were performed, but we prefer the on-label intravitreal injection treatment.

Surgery was performed to treat OS complications when the inflammatory process subsided for almost 3 months, as suggested in the literature. Cataract, the most frequent complication (91.3% of the eyes), was treated with phacoemulsification and intra-ocular lens implantation, resulting in very good visual recovery. Intravitreal Ozurdex was implanted some weeks before cataract surgery in order to prevent post-surgical inflammation and CME in several eyes. We hypothesize that the low rate of complications is due to the good suppression of inflammation in the pre- and peri-surgical period.

Glaucoma complicated the course of the disease in about 37.7% of our cases (89 patients. Trabeculectomy with the use of Mitomycin-C was performed in 54 eyes (11.7%) and it was successful in 44 eyes. Ten eyes had a progressive deterioration of vision because of uncontrolled IOP. Three patients underwent Baerveldt valve surgical implantation, but all of them became blind due to hypotonic complications.

Epiretinal membranes were frequently found in our series (28.8% of the eyes) probably bound to the high inflammatory condition of these eyes. Pars plana vitrectomy successfully treated 48 eyes (10.4%), while the remaining eyes did not require surgery as the membrane was not so severe.

Thirteen eyes (2.8%) underwent cycles of anti-VEGF (Ranibizumab, Lucentis) therapy as treatment of macular CNV complication.

Despite our Centre being a referral department with a cohort of specialists taking care of patients affected by uveitis, with a long-term experience on this specific disease, complications resulted in blindness in at least one eye or legal low vision in 37 patients (15.7%). This suggests that OS is a severe and complex disease, requiring several therapeutical interventions and leading to blindness in some cases, in spite of all the available treatments.

### 4.3. Local and Systemic Therapy

In our study, we followed IWOS recommendations on the management of anterior, intermediate and posterior uveitis in OS [7,9].

Local therapy consists of topical CS administration (0.2% dexamethasone eye drops) several times a day in acute phase of anterior uveitis, then slowly tapered. Topical CS are usually combined with mydriatics; this combination was administered to the majority of patients (85.5%). Mydriatics are useful in preventing posterior synechiae and treating pain bound to ciliary body inflammation.

Sight- or life-threatening disease or CNS with optic nerve involvement requires systemic therapy. Most of our patients underwent systemic CS treatment (203 of 235 subjects, 86.4%) with an initial loading dose of 1–1.5 mg/kg/day then slowly tapered over a few months. The switch from local or intravitreal to systemic treatment was mainly linked to a severe intra-ocular inflammation and to a recrudescence of systemic disease principally affecting the lungs.

More than one-third of patients underwent second-line immunosuppressive therapy with Azathioprine (5.1%), Methotrexate (12.3%), or Mycophenolate Mofetil (18.3%). This was decided given CS side effects, namely, systemic or ocular (IOP) increase, intolerance, uncontrolled systemic, or ocular disease. None of the patients received systemic cyclosporine or tacrolimus therapy.

Less than 10% of patients needed an add-on treatment with other biologic agents (Interferon-alfa 2a, Adalimumab, and Rituximab) as third-line therapy. Systemic treatments were decided by the immunologist who followed our patients both for systemic and ocular sarcoid involvement. Perez-Alvarez et al. demonstrated that biological treatment may itself have the side-effect of inducing both S and granulomatous uveitis [247]. Therefore, these kinds of treatments should be cautiously administered in OS patients. Due to the low number of cases in the literature, they should be further tested in randomized studies in order to be used routinely in the treatment of chronic forms of sarcoid inflammation.

Our retrospective ten-year analysis of clinical data in a homogeneous Caucasian population affected by biopsy-proven or “presumed” OS showed that a multidisciplinary approach finalized to a tailored treatment is useful in limiting severe complications bound to the chronic course of the disease. More controlled studies are needed to give indications for the best therapy and its duration over time.

At the last follow up visit, our patients showed good visual outcomes and a very low incidence of mortality (three patients, 1.3%). We observed a low rate of chronic systemic (42 patients, 17.9%) and ocular (82 patients, 34.9%) sequelae, as expected from the long-term follow-up.

## 5. Conclusions

OS is a diagnostic challenge, especially if systemic symptoms are absent. Even if S remains a disease of unknown etiology, the mechanisms underlying granuloma formation, including genetic susceptibility and environmental factors, are now better understood. Early recognition and therapy are essential to obtain the reduction of systemic and ocular morbidity and for improvement of the patient’s quality of life. Despite the tendency to chronicity of the disease, if medical or surgical treatment is adequate and established early enough, patients have a good prognostic outcome. Moreover, long-term treatment with drugs which potentially produce side-effects and the frequent intra-ocular surgeries make OS a challenging disease.

## Figures and Tables

**Table 1:** The main OS epidemiological studies in recent literature showing sex, mean age, prevalence of ocular involvement and type of uveitis (Anterior Uveitis: AU; Intermediate Uveitis: IU; Posterior Uveitis: PU).

Study	Year of Publicat. (Nation)	Sex (%) F	Mean Age (Year)	Prevalence of Ocul. Invol. (%)	Type of Uveitis (AU IU PU) (%)
Heiligenhaus A. [55]	2011 (Germany)	60.4	34.1	21.2	AU 76.4 IU 17.3 PU 4.7
Yanardagh A. [59]	2018 (Turkey)	55.8	46.2	46.5	AU 35 PU 25
Choi S.Y. [60]	2018 (Korea)	81.8	53.6	70.9	AU 30.8 IU 25.6 PU 43.6
Ungprasert P. [58]	2019 (Usa)	78	51.8	7–15	AU 71 IU 21 PU 7
Dammacco R. [57]	2020 (Italy)	64	53.3	28.7	AU 36 IU 9.1 PU 21

**Table 2:** Systemic therapies for OS complications and posterior involvement (drug, mechanism of action, dosage, way of administration, onset of anti-inflammatory action, main side-effects) [101,199].

Medication	Mechanism of Action	Dose Administration	Onset of Action (Weeks)	Main Side Effects
Steroids	Blockage of inflammatory cascade (COX_2_ inhibition)	0.5–1 mg/kg/day (oral–iv)	1–4 days	Hypothalamic-pituitary-adrenal axis suppression Growth suppression Hirsutism Hyperglycemia Osteoporosis Immunosuppression Cataracts Glaucoma, Psychiatric disturbances
Azathioprine	Alteration of purine metabolism	1–4 mg/kg/day (oral)	4–12	Nausea and vomiting. Loss of appetite Blood in the urine Fatigue Mouth sores and ulcers
Methotrexate	Inibition of hydrofolate reductase	7.5–25 mg/kg/week (oral–im)	2–12	Dizziness Drowsiness Headache Decreased appetite Hair loss
Mycophenolate Mofetil	IMP-dehydrogenase inhibitor	0.5–1.5 Mg twice daily (oral)	2–12	Nausea/Vomiting Diarrhea Urinary Tract Infection Muscles/joints pain Headache
Cyclosporine A	T-cell inhibitor	2.5–10 mg/kg/day twice daily (oral-Iv)	2–6	Hypertension Irsutism Tremors Nausea, diarrhea, Headache Hyperglicemia
Tacrolimus	T-cell inhibitor	0.15–0.30 mg/kg/day (oral-im)	2–6	Headache Diarrhea Nausea/Vomiting Decreased appetite
Infliximab	Anti-TNFɑ	5 mg/kg/day (iv)	1–8	Headache, nausea, skin rash, fever shortness of breath
Adalimumab	Anti-TNFɑ	40 mg/2 weeks (sc)	2–6	Injection site reactions Increased risk of infections Skin rashes
Golimumab	Anti-TNFɑ	50 mg/4 weeks (sc, iv)	1–2	Upper resp. tract infections Injection site reactions Joint pain Viral infections
Certolizumab	Anti-TNFɑ	400 mg/4 weeks Maintenance (sc)	6–12	Same above
Tocilizumab	IL-6 receptor-antagonist	4 mg/kg/4 weeks (iv) 162 mg/1–2weeks (sc)	4	Same above
Interferon ɑ 2a	Antiviral cytokine	3–6 million IU (different regimens) (Im, iv, sc)	24–48 h	Autoimmune diseases Anemia Flu-like syndrome Psychiatric symptoms
Rituximab	Anti-CD20 antigen	500 mg or 1 g each 2 first weeks, then every 16–24 weeks (Iv, infusion)	6–8	Fever chills Anemia Muco-cutaneous reactions Infections Cold symptoms

Evaluate patient before beginning and periodically along treatment course for active TB and test for latent hepatitis B viral infection.

**Table 3:** Demographic characteristics of our cohort.

Demographics	No. of pts	%
Patients	235	-
Eyes	461 eyes	-
Ethnicity: Caucasian Others	221 14	94 6
Sex: Females Males	158 77	67.2 32.8
Definite OS Presumed OS	172 63	73.2 26.8
Bilaterality (eyes)	393 eyes	85.2

**Table 4:** Characteristics of systemic involvement in OS.

	No. of pts	%
Previously diagnosed S	35	14.9
Systemic diagnosis at onset of OS	120	51
Late Diagnosis	52	22.1
No Systemic involvement	28	11.9

**Table 5:** Biopsy, BAL, and laboratory findings.

**Biopsy Site**	**No. of pts**	**%**	
Total	172	73.2	
Lungs	94	54.6	
Lymph nodes	142	82.5	
Skin	59	34.3	
Liver	42	24.4	
Lacrimal Gland	27	15.7	
**BAL Findings**	**No. of pts**	**%**	
Patients	235	45.5	
Positive	72	67.3	
Not specific	35	32.7	
**Laboratory Exams**	**No. pts**	**Increased Levels**	**%**
ACE	235	66	28
Lysozime	83	33	39.7
Calcemia	235	121	51
Calciuria	235	127	5
S-IL-2R	43	36	82

**Table 6:** Radiodiagnostic and nuclear medicine imaging.

^18^F-FDG-PET/CT	No.	%
Patients	183	77.9
Active disease	99	42.1
Thoracic localization	34	14.5
Extra-Toracic localization	11	4.7
Combination	54	22.9
**Chest HRCT Findings**	**No.**	**%**
Patients	215	91.5
Hilar and/or Mediastinic Lymphadenopathy	142	60.4
Parenchimal involvement	94	40
Combination	31	13.2
Negative	16	6.8

**Table 7:** Local and systemic treatment modalities in our cohort.

Treatment	No. pts/Eyes	% pts
Topical (CS+mydriatics)	201/398	85.5
Intravitreal implant	62/94	24.3
Anti-VEGF	3/3	1.3
Sistemic CS	203	86.4
CS+Azathioprine *	12	5.1
CS+Methotrexate *	29	12.3
CS+Mycophenolate M. *	43	18.3
CS+Adalimumab *	11	4.7
CS+Rituximab *	3	1.3
CS+Interferon α-2a *	4	1.7

* CS were used only in the induction phase, then slowly tapered.

**Table 8:** Localization of the ocular disease at onset and ocular complications.

Localization	No. pts	%
Orbit/Adnexa/Sclera/Conjunctiva	21	8.9
Anterior uveitis	112	52.3
Intermediate uveitis	22	10.3
Posterior uveitis	62	28.9
Panuveitis	18	8.4
**Ocular Complications**	**% Eyes (461/100)**	**Eyes**
Synechiae	19.3	89
Cataract	91.3	421
Glaucoma	11.7	54
CME	31.9	147
Epiretinal Membrane	28.8	133
CNV	2.8	13

**Table 9:** Incidence of S among uveitis patients from previous studies modified from Cimino [39].

Country	Sarcoidosis (%)
USA Rodriguez (1982–1992)	9.6
Switzerland Tran (1990–1993)	5.9
UK Jones (1991–2013)	9.7
France Bodaghi (1991–1996) Guillaud (2016)	6.4 15.6
Austria Barisani-Asenbauer (1995–2009)	3.2
Tunisia Khairallah (1992–2003)	1.7
Saudi Arabia Al Dhahri (1998–2013)	4.4
Germany Grajewski (2001–2006) Jacob (2012–2013)	4.5 11.0
Turkey Kazokogu (2004)	0.9
Spain Llorenc (2009–2012)	3.0
Japain Nakahara (2010–2012)	9.4
Italy Cimino (2013–2015) Cimino (2002–2008) Mercanti (1986–1993) Pivetti-Pezzi (1986–1993)	4.3 2.2 0.8 0.2

## Data Availability

Not applicable.

## Methods of Literature Searching

NCBI was the database used and the search words used were “sarcoidosis, ocular sarcoidosis, multimodal imaging, granulomatous uveitis, sarcoidosis-associated uveitis, ocular sarcoidosis diagnosis, ocular sarcoidosis therapy, ocular sarcoidosis complications, anterior uveitis, intermediate uveitis, posterior uveitis, panuveitis, International Workshop on Ocular Sarcoidosis (IWOS), Standardization of Uveitis Nomenclature (SUN), ACE, lysozyme, S-IL-2R, HRCT, Bronchoalveolar Lavage (BAL), ^67^Gallium Scintigraphy, ^18^F-FDG-PET, biopsy in sarcoidosis”. Years covered were 2000–2022 and additional sources (e.g., articles cited in the reference lists of other articles) were not used. The foreign literature was not excluded, and for non-English articles, English abstracts were used. Articles were selected according to journal relevance.
